# Biological Pretreatment Strategies for Second-Generation Lignocellulosic Resources to Enhance Biogas Production

**DOI:** 10.3390/en11071797

**Published:** 2018-07-09

**Authors:** Andreas Otto Wagner, Nina Lackner, Mira Mutschlechner, Eva Maria Prem, Rudolf Markt, Paul Illmer

**Affiliations:** Department of Microbiology, Universität Innsbruck, Technikerstraße 25d, A-6020 Innsbruck, Austria

**Keywords:** pretreatment, biological pretreatment, anaerobic digestion, biogas, methane

## Abstract

With regard to social and environmental sustainability, second-generation biofuel and biogas production from lignocellulosic material provides considerable potential, since lignocellulose represents an inexhaustible, ubiquitous natural resource, and is therefore one important step towards independence from fossil fuel combustion. However, the highly heterogeneous structure and recalcitrant nature of lignocellulose restricts its commercial utilization in biogas plants. Improvements therefore rely on effective pretreatment methods to overcome structural impediments, thus facilitating the accessibility and digestibility of (ligno)cellulosic substrates during anaerobic digestion. While chemical and physical pretreatment strategies exhibit inherent drawbacks including the formation of inhibitory products, biological pretreatment is increasingly being advocated as an environmentally friendly process with low energy input, low disposal costs, and milder operating conditions. Nevertheless, the promising potential of biological pretreatment techniques is not yet fully exploited. Hence, we intended to provide a detailed insight into currently applied pretreatment techniques, with a special focus on biological ones for downstream processing of lignocellulosic biomass in anaerobic digestion.

## 1 Introduction

Although it is known that CO_2_ production from fossil fuel combustion is a major contributor to global warming, these energy carriers are still the most important resources for global energy generation [1]. Great efforts have been devoted to increasing energy production from nonfossil fuels and to replacing climate-change-relevant energy sources by renewable ones. Hydropower, wind, and solar energy are probably the most promising alternative energy resources but can exhibit limitations concerning flexible energy production, storage and/or backup, transportation, and land requirements [2].

Biogas production from anaerobic digestion (AD) processes is considered as an attractive source for green energy [3,4] and, therefore, endeavors have been made to increase the share of biogas in global energy production. During anaerobic digestion, organic feedstocks are converted into biogas containing methane (CH_4_) as a valuable end-product. The energy input for biogas production is calculated to be lower than in current ethanol production, leading to a higher energy output-to-input ratio [5]. However, the expanded production of biogas was often achieved by the utilization of energy crops directly competing with food crop farming (first-generation biofuels). Therefore, the exploration of lignocellulosic materials (second-generation biofuels) for bio-methane production was substantially accelerated during the past years, thus offering ecological as well as economic advantages [6]. However, lignin resists (complete) degradation under anaerobic conditions, posing a challenge regarding the overall degradability of lignocellulose in AD. In this context, enhancing the substrate conversion to overcome the degradation resistance of lignocellulosic resources is of utmost importance to achieving environmentally friendly and economically feasible processes [7,8]. Hence, effective pretreatment methods are needed, particularly because lignocellulosic biomass has been evaluated as an attractive renewable energy source due to its inexhaustible, ubiquitous character [2,9].

The main objectives of this work are, therefore, (i) to present a short update on the currently available pretreatment strategies for enhanced disintegration of lignocellulosic resources and their application, and (ii) to review biological pretreatments currently applied for enhanced biogas production.

## 2 Lignocelluloses

Lignocellulose is the most abundant renewable biomass [10], with a worldwide annual production of an estimated 1000 Gt [11], including wheat straw, sugarcane bagasse, corn stalks, rye straw, rice straw, and barley straw as well as various types of organic waste (fractions). For data on the composition of different feedstocks, please refer to, e.g., Dahadha et al. [12], or Paudel et al. [13].

Lignocellulose contains up to 45% cellulose as the main component, 30% hemicellulose, and 25% lignin, although the composition varies considerably among different plants [14,15]. With about from 50% to 80% of organic material deriving from photosynthetic processes, lignocellulose represents one of the main components of global biomass [16,17]. Therefore, lignocellulose plays a major role as a constituent of biological resources and represents the most abundantly available raw material for the generation of renewable primary products and energy [18].

In the following section the chemical structure and characteristics of the most important fibrous components of lignocellulosic resources are described briefly.

### 2.1 Cellulose

Cellulose is the major component of plant matter and, therefore, a valuable source of biomass storing an enormous quantity of energy conserved by photosynthesis. It is a fibrous, hard, and water-insoluble substance that can be found in the wooden part of plant tissue. As a linear polymer, it comprises from 3000 to 14,000 glucose monomers, which are linked via β-1,4 glycosidic bonds. Approximately 60–70 of those cellulose polymers are interconnected by hydrogen bonds, forming so-called elementary fibrils, which themselves build up microfibrils. Multiple of those collocated chains can form a network of stable supramolecular fibers, with high tensile strength and a partially crystalline structure [19]. In plants, cellulose molecules are synthesized individually, which then undergo immediate self-assembly [20] probably regulated by hemicelluloses [21]. An important feature of cellulose is its crystalline structure, with the degree of crystallinity being highly variable depending on the type of plant tissue [19]. While the crystalline structure of cellulose fibers hinders degradation, various types of irregularities—pits, pores, and capillaries—increase the surface area of cellulose molecules [22]. This results in at least partially hydrated areas when being immersed in water, thus permitting access for enzymatic attack, including cellulases.

### 2.2 Hemicellulose

Hemicellulose is the term for branched heteropolysaccharides—mostly matrix polysaccharides—including monomers like glucose, mannose, galactose, xylose, and arabinose. Although similar enzymes are involved in cellulose and hemicellulose decomposition, complete hemicellulose degradation requires more enzymes due to its greater chemical and structural heterogeneity [23]. Hemicellulose is degraded to monomeric sugars and acetic acid [18], with the latter being of special interest for anaerobic digestion, representing the dominant methane precursor [24].

### 2.3 Lignin

Lignin is an aromatic polymer synthesized of phenylpropanoid precursors. Lignin is predominantly found in combination with cellulose (and hemicellulose), the so-called lignocellulose. Therein, lignin is encrusting both cellulose and hemicellulose, forming a physical seal, and is an impenetrable barrier in the plant cell wall. This polymer is synthesized by the generation of free radicals, which are released in the peroxide-mediated dehydrogenation of three phenylpropionic alcohols [18]. Lignin breakdown is necessary to facilitate the access to cellulose and hemicellulose but can, however, only occur via co-metabolism [18].

## 3 Biodegradation of Lignocellulose

Bioconversion of lignocellulosic residues is predominantly carried out by fungi. Because of the insolubility of cellulose, hemicellulose, and lignin, it occurs exocellularly, in association with the outer cell envelope, or extracellularly [18]. Two types of fungal enzymes are known to break down lignocellulose: (i) the hydrolytic system that produces hydrolases responsible for polysaccharide degradation and (ii) a unique oxidative and extracellular ligninolytic system degrading lignin by opening phenyl rings [18,25].

The ability to digest cellulose is widely distributed among many genera in the domain of Bacteria and in fungal groups within the domain of Eukarya [26], whereas cellulolytic organisms in the domain of Archaea have not (yet) been identified. Specialized groups of fungi are further able to attack lignin-encrusted cellulose. Generally, a 10- to 100-fold higher productivity of fungal compared with bacterial enzymes was assessed for cellulases [25].

Concerning the eubacteria, the ability to decompose cellulose is widespread in bacteria within the predominantly aerobic order Actinomycetales (Actinobacteria) and the anaerobic order Clostridiales (Firmicutes) [19,27]. Mechanisms of bacterial decomposition differ significantly from those of their fungal counterparts. Within cellulolytic clostridia, the breakdown of cellulose is organized in the so-called cellulosome [28,29], which is attached to the cell surface, contains all necessary enzymes, and forms a bridge between the cell and the insoluble cellulose components [16]. In anaerobic digestion systems, cellulose-degrading bacteria play an important role regarding the interaction between several groups of organisms, resulting in a complete conversion into carbon dioxide, methane, and water [30]. However, due to the small amount of energy that can be preserved in anaerobic processes and the lower productivity of bacterial cellulases compared with fungal ones [25], the degradation of cellulose is significantly slower under anoxic than under oxic conditions.

A specialized group within the Neocallimastigomycota called “anaerobic fungi”, commonly found in ruminants, is able to degrade cellulose and hemicellulose under strictly anaerobic conditions [31,32]. The use of anaerobic fungi for an improved anaerobic digestion was taken into account, e.g., by Dollhofer et al. [33] or Leis et al. [34]. Also, Nakashimada et al. [35] investigated methane production from cellulose as a substrate with defined mixed cultures using the cellulolytic *Neocallimastix frontalis* and methanogens.

In contrast to anaerobic fungi, the direct application of aerobically growing fungi in anaerobic systems is completely hampered by their oxygen demand. Among fungi, there are a number of representatives, e.g., of the genera Fusarium and Chaetomonium that also target lignin-encrusted cellulose. In particular, so-called white rot fungi can effectively degrade lignin using an oxidative process with phenol oxidases as key enzymes [36], including *Phanerochaete chrysosporium* and *Trametes versicolor*, representing the most extensively studied members [37]. As the degradation of lignin is hardly possible under anoxic conditions, aerobic pretreatment prior to anaerobic digestion is of special interest [38–40].

## 4 Concepts of Pretreatment

Pretreatment strategies commonly comprise physical, chemical, and biological methods [40], and are applied in various fields of bioenergy and biofuel generation including biogas, bioethanol, biohydrogen, and hythane (H_2_ + CH_4_) production. Since lignocellulose materials represent the largest fraction of waste generated by modern society, increasing scientific interest is orientated towards combined cellulose waste management and energy resources [41]. Factors for ecological and economical feasible application of pretreatment strategies include low capital and energy investments, applicability over a wide variety of substrates, and high product yields to enhance revenues along with low waste treatment costs [7].

Among all bioconversion technologies for energy production, anaerobic digestion seems to be the most cost-effective that has been implemented worldwide for commercial production of electricity, heat, and compressed natural gas [40]. Anaerobic digestion has been adopted for bioenergy production from different organic feedstocks, such as forestry and agricultural residues, animal manures, organic fractions of municipal solid wastes, food wastes, and energy crops [42], answering the increasing demand for renewable energy sources. For recalcitrant substrates such as lignocellulosic resources, conventional anaerobic digestion cannot maximize the substrate conversion into biogas [43]. Thus, the application of biological pretreatments has gained significant importance in the past few years because of (i) the complex composition of lignocellulosic resources persistent in anaerobic environments; (ii) the desire to reduce hydraulic retention times; and (iii) the wish to increase the net carbon conversion rates. The latter is characterized by an enhanced total biogas and methane yield, representing the ultimate goal for any pretreatment strategy.

### 4.1 Physical and Chemical Pretreatment

Physical and chemical pretreatments are the most widespread strategies to improve the substrate quality designated for anaerobic digestion. They are often designed to improve the general digestibility and do not specifically target a certain compound of the substrate matrix. Physical strategies comprise comminution, heat and/or pressure treatment, steam explosion, liquid hot water, extrusion, and irradiation as well as ultrasonic and microwave technologies. Chemical ones include the use of acids or bases, catalyzed steam explosion, ozonization, oxidation, organosolve methods, and ionic liquid extraction [40]. A combination of physical and/or chemical methods is often applied. The main disadvantages of physical/chemical pretreatments are high energy and/or chemical demands with possible quality reductions of the digestion residues, thus hampering the subsequent use as biological fertilizer, accompanied by increasing costs for their disposal [44,45]. For a detailed description of physical and chemical pretreatment strategies, please refer to the respective review papers.

### 4.2 Biological Pretreatment

An effective biological pretreatment requires no preceding mechanical size reduction, preserves the pentose (hemicellulose) fractions, avoids the formation of degradation products that inhibit growth of fermentative microorganisms, minimizes energy demands, and limits costs. Therefore, a major objective of biological pretreatment is to break and remove the lignin seal and to disrupt the crystalline structure of cellulose to make it (more) susceptible to an enzymatic or microbial attack, while minimizing the loss of carbohydrates for anaerobic digestion [40,46,47]. The delignification and the decomposition of hemicellulose enhance the availability of cellulose and resultant monomers, which can boost the overall anaerobic digestion process. The choice of application is mainly dependent on the chemical composition of the substrate; however, in practice, structural and economic factors like available facilities or excess energy can often play an equally important role. Biological pretreatment techniques for enhanced biogas production have mainly focused on the use of fungal and bacterial strains or microbial consortia under both aerobic and anaerobic conditions, as well as on enzymes, with the latter being less important [40]. Therefore, this review is focusing on pretreatment strategies using active microorganisms.

The advantages of biological pretreatments compared with nonbiological procedures are the potential production of useful by-products, reduced formation of inhibitory substances due to milder operation conditions, the minimization of applied chemicals and energy input, and lower costs for waste deposit [44,45]. Beside the hydrolysis of (ligno)cellulose during pretreatment, microorganisms can further be used to upgrade the quality of certain substrates by removing undesired, potentially inhibitory substances. However, the efficiency of biological pretreatment is limited by the rate of microbial growth and the utilization of readily available sugars by the engaged organisms [48].

#### 4.2.1 Micro-Aerobic Pretreatment

Micro-aeration during anaerobic processes is known to increase microbial activity during the initial hydrolysis phase [49]. Pretreatments using different doses of oxygen during anaerobic digestion can also be ascribed to biological pretreatments since the oxygen input alters the microbial community; however, these methods are mainly applied in waste water treatment plants [49,50]. The goal of micro-aeration is to stimulate microbial growth and activity during hydrolysis, the rate-limiting step in anaerobic digestion [51]. Up to now, this method has been successfully applied for brown water and/or food waste [52–54] as well as for energy crops [43] and agricultural residues [55,56].

#### 4.2.2 Ensiling, Composting

Another microbiological pretreatment strategy originated from the necessity to store and stabilize lignocellulosic resources to guarantee a whole year’s substrate supply for anaerobic digestion facilities. Cui et al. [57] investigated a wet storage technique via ensiling with simultaneous chemical and fungal pretreatment which could increase glucose and xylose yields 2.9- and 3.9-fold, respectively. Sugar beet pulp silage in different maturity stages was evaluated concerning its methane potential by Heidarzadeh et al. [58], indicating a positive trend but also the risk of energy loss if the ensiling was not conducted properly. Papinagsorn et al. [59] successfully tested ensiled napier grass in combination with chemical pretreatment for co-digestion with cow dung yielding up to 8.34 kJ·g^−1^ VS. Vervaeren et al. [60] investigated maize silage additives for enhanced biogas production and could verify up to a 14.7% increase in biogas production for certain additives. Wagner et al. [61] used composting as a treatment strategy to enhance methane production from digestate and showed a positive impact of composting with increased biogas and methane yields in a subsequent anaerobic digestion process.

#### 4.2.3 Physical Separation of Digestion Phases or Microbial Consortia

Efforts have been made earlier to separate the different phases of anaerobic digestion to increase total biogas and methane yields. These methods are often referred to special digestion systems but not to pretreatment technologies. Since almost all methods are at least some kind of upstream treatment prior to anaerobic digestion per se, they can also be seen as pretreatment methods. As this type of technology does not aim to increase degradation rates of one or more specific components of substrates, the mode of action is rather unspecific. The physical separation of the hydrolytic and methanogenic phase can further be used to apply suitable conditions (e.g., temperature, pH) for each step. Quin et al. [62] investigated the effect of a thermophilic and hyperthermophilic anaerobic treatment prior to mesophilic anaerobic digestion. In this context, a preceding hyperthermophilic step increased the hydrolytic activity of the engaged microorganisms and resulted in a higher organic solids reduction rate [63,64]. Thermophilic or hyperthermophilic conditions are further beneficial to pathogen removal [65–67]. During aerobic hyperthermophilic pretreatment, *Geobacillus stearothermophilus*, for instance, turned out to be important for downstream processing [68].

#### 4.2.4 Aerobic Pretreatment with Defined Fungal Cultures

Due to their powerful enzymatic capabilities, fungi offer great potential for biotechnological application. Lignocellulose-degrading fungi can be classified into white rot, brown rot, and soft rot fungi. White and soft rot fungi are known to attack lignin and, to a certain extent, also cellulose, while brown rots mainly target cellulose [69]. Nevertheless, white rot fungi are the preferred pretreatment organisms as they mandate highly efficient delignification enzyme equipment [70,71], with basidiomycetes being supremely effective [18,72]. The degree of delignification using white rots varies among applied fungi and depends on various factors such as pH and available N sources [70]. In contrast to anaerobic fungi, higher decomposition rates can be achieved due to aerobic conditions, which lead to higher energy yields for the engaged microorganisms and can therefore result in faster turnover rates. However, the application of fungi to pretreat substrates for anaerobic digestion is rather new [70]. Nevertheless, aerobic pretreatment using fungi was described for various species with diverse outcomes and is summarized in Table 1. A comparison concerning the effectivity and efficiency is rather difficult due to different experimental setups, substrates, inocula, inoculation rates, incubation times, and conditions, etc.; however, the table is intended to give a helpful overview. However, in more than 60 percent of the reviewed publications dealing with fungal pretreatment, white rot fungi were applied with pretreatment periods extending over approximately 3–4 weeks, which is notably longer than for other fungal pretreatments. In most cases, different organic waste fractions were used as substrate; to a minor extent, energy crops were also applied.

Liu et al. [73] found a positive impact of pretreatment on methane production potential using forest residues as co-substrate inoculated with ligninolytic *Ceriporiopsis subvermispora*, but the effect was dependent on the basic substrate used. Ge et al. [74] incubated Albizia (silk tree) biomass, a forestry waste, with the same organism and were also able to increase the cumulative methane yield. From sisal (agave) leaf decortication residues pretreated with two fungal strains including *Trichoderma reesei*, an increased methane production was observed [75]. In comparison with other strategies, Take et al. [76] found a positive effect of biological pretreatment with *Cyathus stercoreus* and *Trametes hirsute* on subsequent biogas production using cedar wood chips as substrate, whereas the pretreatment with *Ceriporiopsis subvermispora* positively influenced the methane yield in another study using the same substrate but with a nutritional supplement [77]. Phutela et al. [78] pretreated paddy straw with *Fusarium* sp. and observed decreased lignin and cellulose contents in the substrate and an improved digestibility. However, the application of a facultative pathogenic microorganism seems to be problematic. Wheat straw was incubated with *Polyporus brumalis* BRFM 985, a white rot fungi, and in combination with metal amendment with the result that the treatment positively influenced the methane potential [79]. In another study by Vasmara et al. [80], 4- and 10-week incubation of wheat straw with 7 different fungal isolates was investigated regarding increased methane yields in a subsequent anaerobic digestion step. A positive effect was found enhancing the methane yield by an optimized treatment up to 16% for the 4-week and up to 37% for the 10-week pretreatment. *Pleurotus ostreatus* and *Trichoderma reesei* pretreatment of rice straw resulted in a 120% increase in methane yields in a study by Mustafa et al. [81]. Moreover, Mustafa et al. [82] found increased delignification and methane production from rice straw by pretreatment with the fungus *Pleurotus ostreatus*. Yard trimmings were subjected to a pretreatment using the white rot *Ceriporiopsis subvermispora* [83]; the enhanced methane production was attributed to an increased delignification by the fungus. Mutschlechner et al. [84] inoculated a similar substrate containing high portions of grass and tree cut with *Trichoderma viride* and could secure increased methane production. This organism was also used to pretreat raw bio-waste with a positive effect on the methane production potential of the substrate [85]. *Phanerochaete chrysosporium* was used during solid-stage fermentation of corn stover to successfully enhance methane production in a subsequent anaerobic digestion step [86]. Tisma et al. [87] observed a positive effect of *Trametes versicolor* pretreatment on the biogas productivity of corn silage. Pretreating orange processing waste with strains of *Sporotrichum*, *Aspergillus*, *Fusarium*, and *Penicillium*, Srilatha et al. [88] observed a positive effect on biogas production and biogas potential. Mackul’ak et al. [89] inoculated sweet chestnut leaves and hay with the fungus *Auricularia auricula*-*judae* and observed an increase in methane productivity. Parthiba Karthikeyan et al. [48] found that biological pretreatments are not yet available for food wastes and demand urgent need for further research.

## 5 By-Product Formation

Pretreatment of recalcitrant material enhances the availability of substrates but can also result in the formation of various inhibitory or even toxic substances. While biological pretreatments are less rigid, physico-chemical ones can be problematic. For example, although high glucose yields are achieved by acid treatment of lignocellulosic biomass, this procedure also leads to hydroxymethylfurfural (HMF) formation, one of the most unwanted pretreatment by-products [10,91]. Moreover, toxic and highly corrosive heavy metal ions like copper, nickel, chromium, and iron are released due to acid application [10]. In contrast, biological pretreatments apply milder conditions, tend to be less corrosive, and release fewer inhibitory substances [92]. Inhibitory substances introduced with the initial substrate can even be degraded during biological pretreatment, leading to an increase in substrate quality. In this context, lignocellulose pretreatment for biofuel production using the fungus *Coniochaeta ligniaria* NRRL30616 led to a degradation of various undesired by-products including phenolic compounds, furfural, and HMF along with an assimilation of these inhibitory substances in the cells and/or a release of less toxic intermediates into the liquid phase [93]. In another study, pretreatment of oil palm mill effluent using thermophilic bacteria resulted in the removal of unwanted phenols coevally improving the anaerobic digestion performance [94,95].

Inhibitory substances can have an adverse effect on the engaged microorganisms involved in biological pretreatment and downstream anaerobic degradation [96] or on pretreatment facilities by corrosion [91,97]. Synergistic toxic effects are known for lignocellulosic hydrolysates, meaning that the toxicity of two or more toxic substances combined (on yeasts) can be higher than their sum [98].

However, a more profound knowledge of inhibitory substances is urgently needed for anaerobic degradation processes including ethanol/biofuel production, waste water treatment, and, especially, biogas production.

## 6 Closing Remarks—Conclusions

Various pretreatment strategies—physical, chemical, and biological—have been developed to overcome the inherent resistance of lignocellulose to anaerobic degradation. Biological pretreatment strategies, however, outcompete other pretreatments due to the application of milder conditions, and lower by-product formation and corrosiveness. The variety of applied techniques comprises micro-aerobic treatments, ensiling or composting, the separation of digestion stages, and pretreatments using various fungi. Fungal pretreatments have achieved particular success using various white, brown, and soft rot fungi, or a combination of these. Pretreatment processes applying white rot fungi from the genera Ceripoioposis, Phanerochaete, Fusarium, Trametes, Polyporus, and Pleurotus target cellulose as well as lignin, allowing the use of recalcitrant, second-generation substrates for biogas production. Therefore, biological pretreatment strategies offer great potential to improve the digestibility of different biogas substrates; however, detailed investigations of the mode of action, the application of different substrates, full-scale implementation, and possible by-product formation are still needed.

## Figures and Tables

**Table 1:** Comparison of different fungal pretreatment strategies for enhanced biogas production.

Pretreatment Organism (Type of Fungus 1)	Substrate	Pretreatment Incubation Conditions 2	Additional Information on Fungal Pretreatment Process 3	Anaerobic Digestion Conditions 4	Impact of Pretreatment on Substrate	Impact of Pretreatment on Biogas Production	Reference
*Ceriporiopsis subvermispora* (wrf)	Japanese cedar wood	8 weeks a 28 °C b 70% c	orig, hyphal biomass grown on agar added, substrate supplemented with 10% wheat bran.	batch, mp, t 60	28% lignin removal in initial substrate, ~75%cleavage of ß-O-4 aryl ether	35% and 5% conversion of holocellulose to methane with and without pretreatment, respectively	[77]
*Ceriporiopsis subvermispora* (wrf)	Albizia biomass (forestry waste)	48 days a 28 °C b 60% c	e, autoc	batch, mp, ssAD, t 58	24% lignin removal of initial substrate, 4-fold increase in xylose and glucose production after 72 h of enzymatic hydrolysis	3.7-fold increase in methane production	[74]
*Ceriposiopsis subvermispora* (wrf)	Hazel and acacia branches, barley straw, and sugarcane bagasse	28 days a 28 °C b	e, autoc, grinded substrate	batch, mp	2- to 4-fold increase in enzymatic cellulose degradability for hazel and bagasse, decrease for straw and acacia	Increase of biomethane potential (BMP) for hazel (60%), loss of BMP for acacia (34%), straw and sugarcane bagasse	[73]
*Phanerochaete chrysosporium* (wrf)	Corn stover silage	30 days a 28 °C b Stable ambient d	f, autoc, washed substrate	batch, mp, t 30	39% lignin removal of initial substrate, improved degradation of substrate cell wall components	19.6–32.6% increase in methane production compared with controls	[86]
*Fusarium* sp. (wrf)	Paddy straw	10 days a 30 °C b 70% c	g, orig	batch, mp, t 35	17.1% decrease in lignin content, 10.8% decrease in silica content compared with controls	53.8% increase in biogas production	[78]
*Trametes versicolor* (wrf)	Corn silage	7 days a 27 °C b 70–80% c	g, orig	cont, mp, co-digestion with cow manure	70% increase in lignin degradation compared with control approach	Increased pH stability and biogas productivity, enhanced anaerobic degradation	[87]
*Ceriporiopsis subvermispora* (wrf)	Yard trimmings	30 days a 28 °C b 60% c	e, autoc	batch, mp, ssAD, t 40	20.9% degradation of initial lignin content	54% increase in methane production compared with controls, increased cellulose degradation	[83]
*Polyporus brumalis* (wrf)	Wheat straw	12.5 to 20 days a 20–30 °C b wet weight to initial solid ratio of 2.1 to 4.5	e, autoc, addition of metal supplement solution	batch, mp, t 57		Decrease in methane production compared with the control. Within fungal pretreatment, best methane production after 12.5 days incubation at 30 °C at 3.7 ww/ts ratio	[79]
*Pleurotus ostreatus* (wrf) *Trichoderma reesei* (srf 5)	Rice straw	20 days a 28 °C b 75% c	g, autoc	batch, mp, ssAD, t 45	33% lignin removal of initial substrate with wrf and 23.6% with brf Lignin-to-cellulose ratio after treatment: wrf 4.2, brf 2.88	20% increase in methane production with wrf and 21.7% decrease for brf treatment	[81]
*CCHT-1* (wrf) *Trichoderma reesei* (srf 5)	Sisal leaf decortication residues	4 + 8 days a 28 °C b	g, orig, two fungal stages: wrf followed by brf	batch, mp, t 84	22.5%. decrease in neutral detergent fiber content, 21% increase in cellulose content	30–101% increase in biogas production compared to control	[75]
*Sporotrichum* sp. *Aspergillus* sp. *Fusarium* sp. *Penicillium* sp.	Orange processing waste	3 days a 30 °C b 65% c	g, orig, mixed culture pretreatment.	cont, mp, t 25	Reduction in inhibitory limonene content in the substrate.	Pretreatment leads to higher possible organic loading rates that improve overall productivity	[88]
*Trichoderma viride* (srf 5)	Organic waste	4 days a 25 °C b	e, orig	batch, tp, t 18	Increased cellulase activity during pretreatment compared with controls	Up to 400% increase in methane production compared with controls	[85]
*Trichoderma viride* (srf 5)	Organic waste	10 days a 22 °C b 70% c	f, orig	batch, tp, t 14	Increased cellulase and dehydrogenase activity compared to control	More than 2-fold increase in methane production	[84]

1 **wrf**: white rot fungi; **brf**: brown rot fungi; **srf**: soft rot fungi

2 **a**: incubation period, **b**: incubation temperature, **c**: moisture content in %, **d**: humidity in %

3 inoculation with **e**: submerged fungal culture, **f**: fungal spores, **g**: autoclaved substrate overgrown by fungal mycelia; autoc: autoclaved substrate, orig: original, unmodified substrate

4 **batch**: batch system, **cont**: continuous system, **ssAD**: solid-state anaerobic digestion, **mp**: mesophilic conditions, **tp**: thermophilic conditions, **t**: anaerobic incubation period or hydraulic retention time in days

5 according to Klein and Eveleigh [90].
